# Effect of an initial specimen diversion device on blood-culture contamination rates and vancomycin usage: A quasi-experimental study

**DOI:** 10.1017/ice.2023.163

**Published:** 2024-01

**Authors:** Eli P. Wilber, Ahmed Babiker, Jessica Howard-Anderson, Jill E. Holdsworth, Eileen M. Burd, M. Jeremy Eldridge, Jesse T. Jacob

**Affiliations:** 1 Division of Infectious Diseases, Department of Medicine, Emory University School of Medicine, Atlanta, Georgia; 2 Department of Pathology and Laboratory Medicine, Emory University School of Medicine, Atlanta Georgia; 3 Office of Quality, Emory University Hospital Midtown, Atlanta, Georgia; 4 Department of Emergency Services, Emory University Hospital Midtown, Atlanta, Georgia

## Abstract

Initial specimen diversion devices (ISDDs) are a potential solution for reducing blood-culture contamination rates. We report the implementation of an ISDD associated with a sustained reduction in blood-culture contamination rates for >18 months after implementation. We did not observe a clinically significant reduction in inpatient vancomycin usage.

Blood cultures remain the primary modality for diagnosing bacteremia and sepsis. The accuracy of blood cultures is hampered by the substantial false-positive rate attributable to contamination with skin flora.^1^ To combat this, devices have been developed that divert the initial aliquot of blood from the culture bottle (ie, initial specimen diversion device or ISDD). These devices have decreased blood-culture contamination rates relative to standard phlebotomy practices in short-term prospective efficacy studies.^2–6^ Also, ISDD implementation may decrease the rate of false-positive central-line– associated bacteremia.^5^ However, data are limited regarding whether implementation of ISDDs can result in a sustained reduction in contamination rates or in decreased antimicrobial use. We hypothesized that ISDD implementation would decrease inpatient vancomycin use as a result of less initial treatment of bacteremia that was ultimately determined to represent contamination.

We present a large, controlled, quasi-experimental study to assess the implementation of an ISDD in the emergency department (ED) on blood-culture contamination rates and vancomycin usage.

## Methods

### Intervention and definitions

We performed a quasi-experimental study in two 500-bed academic hospitals in Atlanta, Georgia. In January 2018, the intervention hospital implemented the use of an ISDD (Steripath, Magnolia Medical Technologies, Seattle, WA) in the ED whereas the control hospital did not. Both hospitals collected and provided information about blood-culture contamination rates at the group and individual levels before and after the intervention. The groups were defined as ED or inpatient groups. Data were collected for 29 months before implementation and 20 months after implementation. Blood-culture contamination was defined as the presence of “skin microbiota” organisms in only 1 of 2 or more blood-culture sets collected within 24 hours (Supplementary Material online).^7^ The blood-culture contamination rate was defined as the number of contaminated cultures divided by the total number of blood cultures. Vancomycin days of therapy (DOT) data were abstracted from antimicrobial stewardship surveillance data.

### Statistical analysis

We calculated median pre- and postimplementation blood-culture contamination rates for each ED and inpatient setting and compared them using the Wilcoxon rank-sum test. An interrupted time series (ITS) analysis using a quasi-Poisson regression model (Supplement S2 online) was used to analyze the impact of the ISDD on contamination rates as well as vancomycin DOT per 1,000 patient days. This analysis was performed using R Studio software (R Foundation for Statistical Computing, Vienna, Austria).

## Results

**Figure 1.**
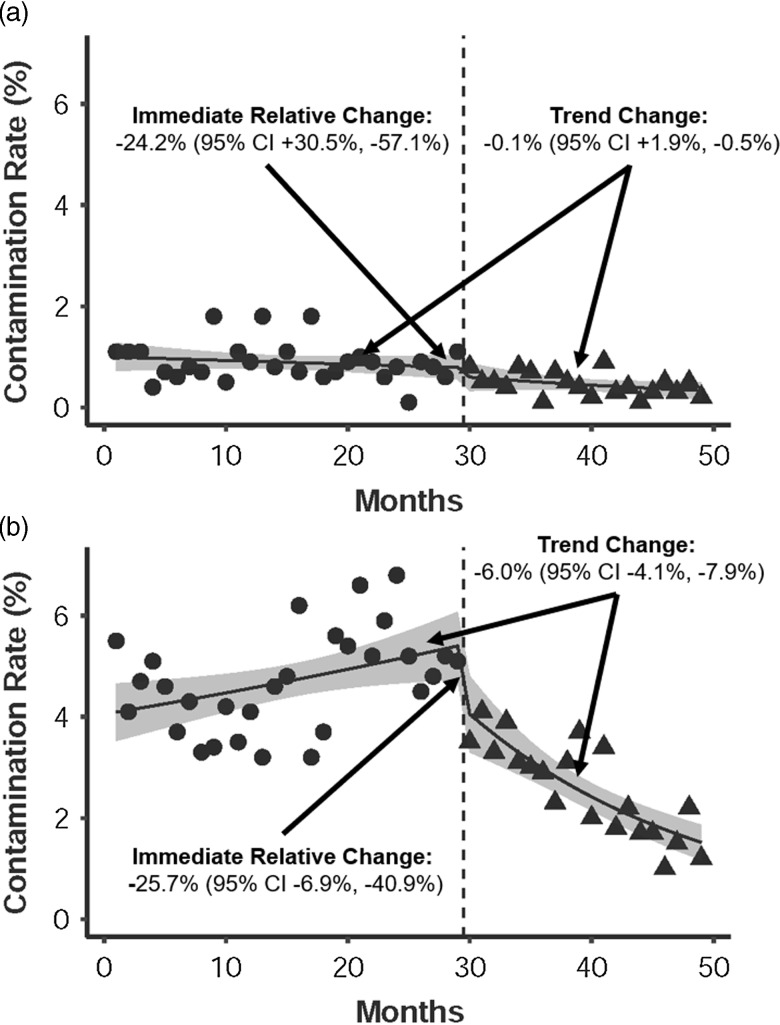
Observed emergency department blood-culture contamination rates before (circles) and after (triangles) the intervention (dashed line) at (A) the control hospital and (B) the intervention hospital. Solid lines represent contamination rates using a quasi-Poisson regression model, and the gray shaded area represents the 95% confidence interval.

Among 191,789 blood cultures, 4,025 (2.1%) were contaminated. At the intervention hospital, the postintervention period had lower median blood-culture contamination rates compared to the preintervention period in both the ED (2.6% vs 4.7%; *P* < .001) and the inpatient setting (0.5% vs 0.8%; *P* < .001). At the control hospital, there was no significant change in ED contamination rates (5.0% vs 5.0%; *P* = .89); however, there was a change in inpatient contamination rates (0.6% vs 1.0%; *P* = .002). In ITS analysis, the intervention was associated with an immediate relative decrease in the intervention hospital’s ED contamination rate by 25.7% (95% confidence interval [CI], −6.9% to −40.9%) (Fig. 1). Additionally, there was a trend change in the intervention hospital’s ED contamination rates of −6.0% per month (95% CI, −4.1% to −7.9%). In contrast, the intervention hospital’s inpatient blood-culture contamination rate had no significant immediate change (−24.2%; 95% CI, +30.5% to −57.1%) or trend change (−2.2% per month; 95% CI, +2.5% to −6.8%). There were no significant immediate or trend changes for the control hospital’s inpatient or ED contamination rates.

**Figure 2.**
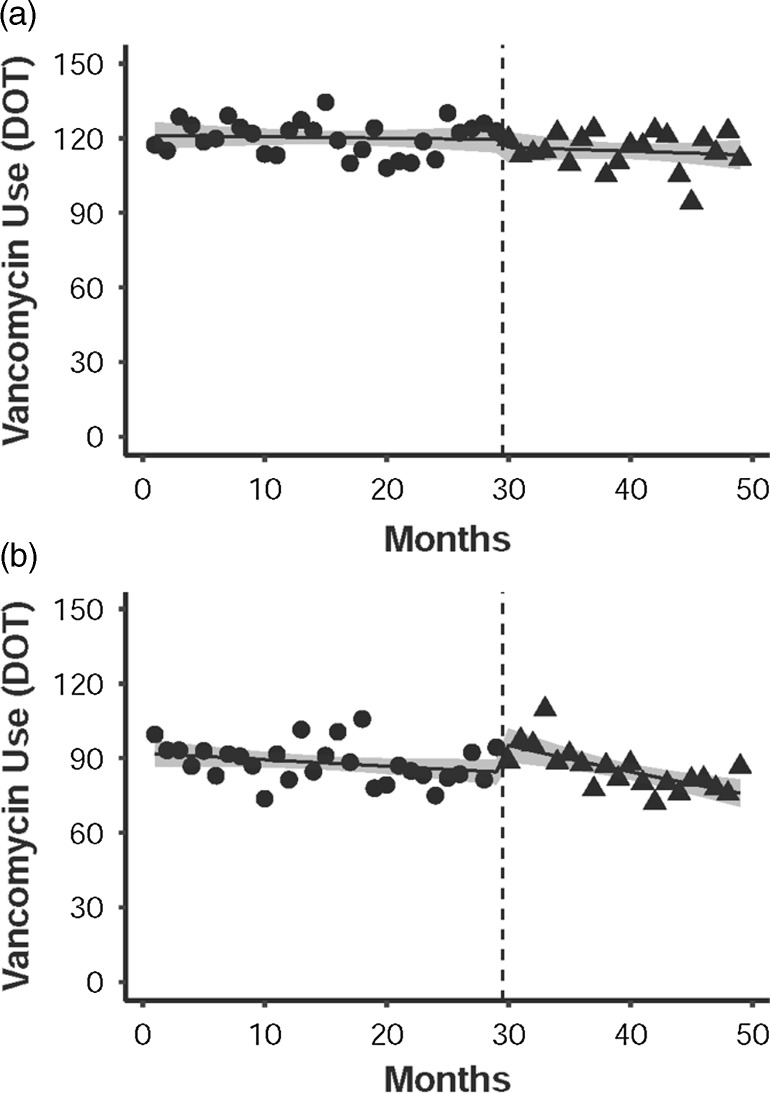
Observed vancomycin usage rates before (circles) and after (triangles) the intervention (dashed-line) at (A) the control hospital and (B) the intervention hospital. Solid lines represent modeled vancomycin usage rates using a quasi-Poisson regression. The gray shaded area represents the 95% confidence interval of the modeled vancomycin usage rate.

At the control hospital, there was no significant level or trend change in inpatient vancomycin use across the study period (Fig. 2). At the intervention hospital, the implementation of the intervention was associated with an immediate increase in inpatient vancomycin use (+10.9 DOT per 1,000 patient days; 95% CI, 2.3–20.4). We observed a trend change in the rate of inpatient vancomycin use after the intervention (−0.9% per month; 95% CI, −0.2% to −1.6%).

## Discussion

We report a 25% immediate reduction in blood-culture contamination rates after ISDD implementation. This reduction persisted, and was further improved upon, during 20 months of follow-up data. Given the size of our study (>190,000 blood cultures) and the duration of follow-up, these findings provide strong supportive evidence that implementation of an ISDD sustainably reduces blood culture contamination rates.

Interestingly, we observed a transient increase in inpatient vancomycin use immediately after the ISDD implementation despite a decrease in the blood-culture contamination rate. This increase was not sustained, and the observed trend change in vancomycin use was small (<1% per month), suggesting a small gradual effect of the intervention on total inpatient vancomycin use. We interpret this finding as showing that blood-culture contamination is a minor driver of total inpatient vancomycin use and that an immediate level change in vancomycin use in response to ISDD implementation is unlikely. A more accurate metric would be vancomycin use in the first 3 days of hospitalization; however, we did not have access to these data. Future studies of ISDDs should evaluate their impact on other outcomes such as length of stay and unnecessary testing (eg, repeat blood cultures).

In contrast to our study, Nielson et al^2^ reported a significant decrease in vancomycin DOT following ISDD implementation. These researchers analyzed the impact of 2 interventions on vancomycin DOT. The first intervention used nucleic acid amplification testing (NAAT)–based microbial identification for blood cultures, and the second intervention, instituted 8 months later, used ISDD implementation. These researchers reported no significant effect of NAAT-based identification on vancomycin DOT, but they did find a significant effect with ISDD implementation. However, ITS analysis was not used, and an alternate hypothesis is that NAAT-based identification required a learning period before the effects on vancomycin usage were realized.

One strength of the ITS design is the ability to model pre- and postintervention trends, which increases confidence in estimating an intervention’s effect.^8^ This methodology is well suited to before-and-after quasi-experimental studies, which are commonly used to evaluate quality improvement initiatives. Additional strengths include the large number of cultures, inclusion of a control group, and the extended postintervention period. The latter allowed us to demonstrate that the reduction in contamination rates associated with ISDD implementation is sustainable beyond a “rollout” period.

This study had several limitations. We were unable to track how often the ISDD was used in the intervention hospital’s ED, which may have led to the underestimation of the effect of the intervention. At both hospitals, feedback on contamination rates was given to unit leadership and individual providers, but we were unable to measure or to control for how this informed action. Additionally, the changing patient case mix could have contributed to the observed differences in contamination rates or the lack of observed difference in vancomycin usage. However, the use of a nearby control hospital with a similar patient and provider population helped mitigate this limitation.

In conclusion, we report the implementation of an ISDD associated with a substantial, sustainable decrease in blood-culture contamination rates but without a major effect on vancomycin DOT. ISDDs have the potential to improve patient care by reducing unnecessary downstream testing that results from blood-culture contamination. Further research is needed to couple this intervention with antibiotic stewardship, to assess the impact on patient-centered outcomes (eg, length of stay), and to quantify the potential financial benefits to facilitate wider adoption of these devices.

## Supplementary material

For supplementary material accompanying this paper visit https://doi.org/10.1017/ice.2023.163. click here to view supplementary material
