# Supplementary material for: Effect of an initial specimen diversion device on blood-culture contamination rates and vancomycin usage: A quasi-experimental study
Source: Infect Control Hosp Epidemiol. 2023 Aug 3;45(1):100–2. doi: 10.1017/ice.2023.163 (PMC10782199; doi:10.1017/ice.2023.163)
Supplement: Supplementary file 1 [file S0899823X23001630sup001.docx]

**Supplemental Material**

**S1.** List of skin microbiota considered to be blood culture contaminants if identified in only 1 of ≥2 blood culture sets collected within 24 hours.

Coagulase-negative Staphylococi

Micrococcus spp.

Viridans-group Streptococci

Propionibacterium acnes

Corynebacterium spp

Bacillus spp

**S2.** Example of quasi-Poisson model used in this study.

ln(Intervention Hospital contamination rate)_T_ = β_0_ + ­ β _1_­*Time + β_2_*pre_post + β_3_*time_after

**Time**= study time measured in months

**pre_post**= dummy variable coding whether time-point is before or after the intervention (0 = pre-intervention, 1 = post-intervention)

**time_after**= time since the intervention measured in months. The month of the intervention implementation and all preceding months are 0. (0….0, 1, 2, 3).

Level change = exp(β _2_)

Trend change= exp(β _3_)
